# Laparoscopic Sigmoid Vaginoplasty for the Treatment of Mayer–Rokitansky–Kuster–Hauser Syndrome in a Single Center: 20 years’ Experience

**DOI:** 10.1007/s00192-024-05829-w

**Published:** 2024-06-13

**Authors:** Lu Yang, Guanghua Xu, Kaixiong Tao, Gang Lv, Zehua Wang

**Affiliations:** 1 Department of Obstetrics and Gynecology, Union Hospital, Tongji Medical College, Huazhong University of Science and Technology, Wuhan, 430022 China; 2 Department of General Surgery, Union Hospital, Tongji Medical College, Huazhong University of Science and Technology, Wuhan, 430022 China

**Keywords:** Mayer–Rokitansky–Kuster–Hauser syndrome, Laparoscope, Sigmoid colon, Vaginoplasty

## Abstract

**Introduction and hypothesis:**

We investigate the feasibility, safety, and clinical therapeutic effect of laparoscopic sigmoid vaginoplasty in women with Mayer–Rokitansky–Kuster–Hauser (MRKH) syndrome.

**Methods:**

We performed a retrospective case review cohort study of 56 patients with MRKHs undergoing laparoscopic sigmoid vaginoplasty in Wuhan Union Hospital between 2000 and 2020, and all patients were followed up.

**Results:**

The median operating time was 165 min (120–420 min). The median hospital stay was 10 days (rang 7–15 days). A functional neovagina was created 11–15 cm in length and two fingers in breadth in all patients. No introitus stenosis was observed. No intra- or post-operative complications occurred. Two patients were lost to follow-up after 3 months of outpatient visits. Six patients had no intercourse and were required to wear a vaginal mold occasionally. None of the patients had complained of local irritation or dyspareunia. Patients who had post-surgery sexual intercourse were satisfied with their sexual life and the mean total Female Sexual Function Index (FSFI) score was 25.17 ± 0.63. The cosmetic results were excellent.

**Conclusions:**

The laparoscopic sigmoid vaginoplasty can achieve the goal of making a functional neovagina. The main advantage of this surgical technique is that it is minimally invasive and that there are fewer complications post-operation. It is an acceptable procedure for patients with MRKH syndrome.

## Introduction

Mayer–Rokitansky–Kuster–Hauser (MRKH) syndrome is a rare developmental disorder of the female reproductive system with an incidence of about 1/5,000–1/4,000 [1]. MRKH syndrome is caused by the abnormal differentiation of the paramesonephric duct during the embryonic period and is one of the most common causes of primary amenorrhea [2]. Patients with MRKH syndrome have normal sexual characteristics and 46 XX chromosomes, characterized by uterine and vaginal hypoplasia in the upper two-thirds. It can be divided into two types according to its clinical manifestations: type I is the simple type, characterized by a primordial uterus and an absent vagina, whereas other systems such as the urinary and skeletal systems develop normally, which is the most common type; type II is also called the complex type, showing malformation or dysplasia of the urinary system, the skeletal system, and other systems, in addition to a bilateral primordial uterus and an absent vagina, about 25—50% of which are combined with urinary system malformation; 10—15% are combined with urinary system malformations and skeletal malformations, cardiovascular malformations, deafness, cleft palate, etc. [3]. Vaginoplasty can be performed in a variety of ways. The surgery is aimed at reconstructing the vagina, solving the patient’s sexual problems, and improving the patient’s quality of life [4]. Many techniques have been used for vaginoplasty, including amniotic membrane, flap, peritoneum, and biological mesh. However, each of these techniques has its limitations, such as stenosis, insufficient length, insufficient lubrication, risk of bladder injury, and high cost. Sigmoid vagina replacement can well imitate the normal vaginal structure in terms of morphology and function and is a relatively common operation at present [5]. In addition, laparoscopic surgery has the advantage of minimal trauma, quick recovery, minor post-operative pain, and an abdominal incision. We hypothesize that laparoscopic sigmoid vaginoplasty might be better than other techniques.

Since 2000, with the introduction of endoscopic instruments and the improvement of surgical techniques in our hospital, we have started to use laparoscopic sigmoid vaginoplasty for patients with MRKH syndrome. We retrospectively analyzed the cases in our hospital over the past 20 years to study the feasibility and effectiveness of this procedure.

## Materials and Methods

### Patients

From January 2000 to December 2020, a total of 56 patients with MRKH syndrome underwent laparoscopic sigmoid transvaginal surgery in our hospital. The patients ranged in age from 15 to 33 years (mean age 23.07 years) at the time of surgery. Pelvic and abdominal ultrasonography, sex hormone determination, and chromosome examination were performed pre-operatively. Of these, 49 had a primordial uterus, and 7 had no uterus. There were 7 patients with urinary malformation, 2 with arrhythmia, and none with cardiac or skeletal malformation. The secondary sexual characteristics of the patient were well developed, and the sex hormone test was normal. Among all the patients, the karyotype of the chromosome in 53 of them was 46 XX, whereas the other 3 showed some differences, with 46X, dir(X), t(x,4), 46XX, 21PS + and 46XX, 15 ps + separately. One patient’s elder sister was a primary uterine patient. One had a left nephrectomy, 2 had inguinal hernia surgery, 2 had breast surgery, and 1 had appendix surgery (Table 1).

**Table 1:** Clinical characteristics of the patients

Patients	Group	Number
MRKH syndrome	Type I	49
	Type II	7 (urinary malformation)
Uterus states	Primordial uterus	49
	No uterus	7
Chromosome examination	Normal	53 (46, XX)
	abnormal	3 (46X, dir(X), t(x,4); 46XX, 21PS + ;46XX, 15 ps +)
Age(years old)	< 18	2
	18–30	52
	> 30	2
Marital status	Unmarried	25
	Married	31
Family history	Yes	1
	No	55
Surgery history	Yes	6
	No	50
Urinary malformation	Yes	7
	No	49
BMI	< 18	7
	18–24	43
	> 24	6
Merkel’s diverticulum	Yes	0
	No	56

*MRKH* Mayer–Rokitansky–Kuster–Hauser, *BMI* body mass index

### Bowel Preparation

All the patients consumed a semiliquid diet for 3 days before the operation. Metronidazole was also prescribed. At 12 h before the operation, the patients received a cleansing enema and intravenous nutrition. Traditional skin preservation of the perineal region was performed before the operation.

### Main Instruments

Laparoscopic instruments and equipment (Stryker), an ultrasonic knife (Johnson & Johnson), and a laparoscopic linear closure cutter (ATB45) and tubular stapler (CDH29) were selected.

### Pre-Operative Preparation

After receiving a general anesthetic, the patients were placed in the lithotomy position (left high right low, Trendelenburg position). In the middle of the umbilicus, a needle was inserted to establish a pneumoperitoneum and maintain a pressure of 14 mmHg. A trocar was then placed on the site, the same procedure as during colorectal surgery. The ureters and ovaries in the abdominal cavity were then examined, and the depth of the pelvic cavity was measured. The length of the sigmoid colon and mesocolon was assessed. The distribution and shape of the arterial branches were observed using transillumination of the mesocolon to determine the length of the sigmoid colon and artery revascularization.

### Operative Method

#### Placement of the Laparoscopic Sigmoid Vaginoplasty Instruments

We established a 13 mmHg (1 mmHg = 0.133 kPa) CO_2_ artificial pneumoperitoneal cavity. We selected four conventional laparoscopic incisions: the upper edge of the umbilicus (10 mm), 3 cm on the right side of the umbilicus (5 mm), the right lower abdominal (5 mm), and the outer third of the line between the umbilicus and the left anterior superior iliac spine (5 mm).

#### Vaginal Cavitation

A transverse incision was made below the urethra between the labia minora. By gentle blunt and sharp dissection, a three-finger-wide space was created dorsal to the urethra and bladder and ventral to the rectum, to reach the pelvic cavity. An ultrasonic knife was used to cut the pelvic floor peritoneum below the primordial uterus.

#### Obtaining the Desired Sigmoid Segment

Laparoscopic display of the descending colon, sigmoid colon, and mesenteric blood supply of the rectum was performed. Proximal and distal tangents were selected according to the depth of the pelvic cavity and the length of the sigmoid mesangial to ensure a 15-cm segment of the intestine. The lateral sigmoid peritoneum was opened using an ultrasonic knife from the tangent line of the proximal rectum, and the anteroposterior mesangium was opened in a fan shape. The sigmoid colon was dissociated to the retroflexion of the pelvic peritoneum, and the main arteries of the mesentery were reserved. The grafted sigmoid colon was obtained by sealing and cutting the proximal and distal ends of the sigmoid colon with a laparoscopic linear closure cutter.

#### Sigmoid Anastomosis

The trocar incision on the right abdominal wall was extended to 2.5 cm, the distal end of the descending colon was pulled out of the abdominal wall incision, a stapler screw was inserted, sutured and fixed, and then returned to the abdominal cavity. The tubular stapler was inserted through the anus to the broken end of the rectum, connected with the screw drill, and sutured distally to the descending colon. The tubular stapler was activated and then withdrawn. The two sides of the intestinal ends cut by the anastomat were in a continuous annular shape.

#### Vaginogenesis

The distal end of the free sigmoid colon was sent into the acupoint and pulled to the outer opening of the acupoint. After the intestinal wall edge was aligned with the vaginal vestibular mucosal edge, the synthetic line was used for intermittent suture.

#### Laparoscopic Examination

The edge of the pelvic peritoneal incision was sutured and fixed around the intestinal segment. It should be checked whether the intestinal mesangium of the sutured, fixed, and free intestinal segment and the anastomosis sites are normal.

### Post-operative Treatment

After the operation, the artificial vagina required a vaginal mold and was washed once with clear water every other day. Routine post-operative care included flushing around the anus twice a day. Prior to the recovery of intestinal function, the patients received intravenous nutrition support. After the passage of gas by anus, the diet of the patients gradually transitioned from a liquid to a semiliquid diet. All the patients were given prophylactic antibiotics, and any changes in abdominal signs were closely observed.

### Follow-up

For the first month after the operation, the patients were followed up once per week by using questionnaires and telephone interviews. During the second month after the operation, the follow-up period was once every 2 weeks. Three months after the operation, all the patients returned to the hospital outpatient department for a face-to-face visit. During the consultation, patients underwent specialized gynecological examination and acquired medical tips and life advice. Thereafter, patients returned to the hospital regularly every 3 months. Three years after the operation, the follow-up period was once every 6 months. The follow-up included assessments of vaginal size, volume, length, and color, as well as vaginal discharge and secretions. The married patients and the patients who had sexual partners were also questioned about aspects of their sex lives.

### Statistical Analysis

The software SPSS 19.0 (IBM) was used to perform the statistical analysis. Values are expressed as the mean ± standard deviation or *n* (%). Statistical analyses were performed using Student’s *t* test for continuous measurement data and Chi-squared test for dichotomous or categorical variables. A *p* value < 0.05 was considered to indicate statistical significance.

## Results

We retrospectively analyzed the patients with MRKH syndrome admitted to our hospital from January 2000 to December 2020. There were nine unoperated patients, 1 of whom did not return to the hospital for surgery after discharge owing to waiting for chromosomal results; 1 patient had a blind vaginal end of about 4.5 cm and was treated using the mold top pressure method; three patients had a false vaginal length of about 6–7 cm, were discharged, and whether they needed surgery depended on their sexual life in the future. Two patients waited for surgery after the age of 18, and 2 patients refused surgical treatment. Among the patients who received the operation, 7 patients underwent abdominal flap vaginoplasty, 2 underwent laparoscopic peritoneal vaginal replacement, and 56 underwent laparoscopic sigmoid colon vaginal replacement. All the procedures were successfully performed with no intra-operative morbidity and no conversion to open surgery (Table 2).

**Table 2:** Intra- and peri-operative outcome: patients who underwent laparoscopic sigmoid vaginoplasty

Surgery types	No	Median operation time (range, min)	Median blood loss (range, ml)	Intra-operative complications	Post-operative complications*
Laparoscopic sigmoid vaginoplasty	56	165 (120–420)	100 (20–2,000)	0	0
Abdominal flap vaginoplasty	9	180 (150–300)	75 (50–200)	0	4 (2 incision infection; 2 vaginal contraction)
Laparoscopic peritoneal vaginal replacement	2	110 (100–120)	125 (100–150)	1 (bladder injury)	0
Non-operative patients	9	–	–	–	–

*p < 0.05

For all the laparoscopic sigmoid colon vaginal replacement patients, the average operative time was 178.15 ± 49.76 min, and the average amount of bleeding was 131.48 ± 267. 95 ml. Owing to the difficulty of vaginal cavitation and teratoma removal at the same time, there was one patient with about 2,000 ml of bleeding. Besides the special case, the blood loss range of the other 55 patients was 20–300 ml. Although there was one patient with intra-operative bleeding and a difficult operation, there were no other major intra-operative complications, and none of the patients was converted to open surgery. The prognosis of the patient was good. The peri-operative complications included one patient with an umbilical incision infection. The hospital stay was 9.45 ± 2.15 days. A functioning vagina was created in all women. The mean length of the neovagina was 13.12 ± 1.14 cm (range, 11–15 cm), and the mean width was 3.99 ± 0.26 cm before discharge (Table 2).

Two patients were lost to follow-up after their 3 months’ outpatient visits. No length shrinkage was observed during post-operative follow-up. Forty-eight patients had subsequent sexual activity. The interval between the operation and first intercourse was 2–6 months. These patients were all satisfied with their body image. From interviews, none of these women developed vaginal stenosis due to the contracture causing painful intercourse or had spontaneous bleeding from the vagina. The other six women still required a vaginal stent every night because they had no sexual partner. Among the sexually active patients, 83.3% (40 out of 48) answered the FSFI questionnaire and completed all items: 28 of the 40 patients (70%) answered during their outpatient follow-up visit and 12 (30%) by telephone. The other 16.7% (8 out of 48) did not answer the FSFI questionnaire; however, during an outpatient follow-up visit, they reported satisfaction with their post-operative sexual intercourse. The mean desire, arousal, lubrication, orgasm, satisfaction, and pain scores were 3.85 ± 0.76, 4.09 ± 0.55, 4.35 ± 0.54, 4.02 ± 0.72, 4.61 ± 0.85, and 4.25 ± 0.36 respectively. The mean total score was 25.17 ± 0.63.

## Discussion

Mayer–Rokitansky–Kuster–Hauser syndrome is the most common disease in the congenital absence of a vagina, whereas androgen insensitivity syndrome is less common. Patients with MRKH syndrome have normal genotype, endocrine status, and ovarian function. Severe dysplasia of the paramesonephric duct is often accompanied by urinary tract malformation. Pre-operative chromosome examination should be performed, and a sex hormone test is recommended to understand endocrine function simultaneously. It is best to perform heart and urinary examination before surgery to determine if there is any developmental abnormality to facilitate the diagnosis and classification of the disease. Proper diagnosis of the underlying disease is important, and these associated deformities should be excluded pre-operatively to avoid surgical injury.

Although the incidence of MRKH is not high, it seriously affects the psychological and social status of patients. The treatment should focus both on the vagina reconstruction and on the function to improve the psychological status of patients. For patients with MRKH syndrome, the treatment methods include non-operative pressure method and surgical treatment, and individual selection should be made according to the patient’s age and needs. The nonsurgical method involves gradual dilatation of the vaginal dimple at the introitus. This requires time and strong patient motivation. As the procedure is painful and self administered, compliance is usually very poor [6, 7]. Surgical treatment is the main method. The development of artificial vaginoplasty has taken more than 100 years, with various procedures, including the amniotic method, the sigmoid vaginoplasty [6, 7], the peritoneal vaginoplasty, and the vestibular mucosal levitation vaginoplasty [6, 8]. These surgical methods have their characteristics and some disadvantages, such as long mucosal formation, long-term vaginal mold use, hair growth, skin flap prolapse, and an obvious scar in the donor area. The sigmoid vagina replacement is a good choice for vaginoplasty, its advantages include: little narrowing after vaginal formation, no need to wear a mold for a long time, the intestinal mucus can play a lubricating role, and the appearance and organizational structure are close to the normal vagina [9]. However, the disadvantages of traditional open sigmoid vaginal replacement surgery limit its extensive development. The most important disadvantages are long operation time, great interference with the abdominal and intestinal tract, gastric tube placement to prevent intestinal obstruction, slow post-operative recovery, and a long hospital stay [10]. In addition, the larger post-operative surgical scar on the abdomen affects the appearance and causes an unnecessary psychological burden to the patients. Laparoscopic surgery significantly reduces the above disadvantages. With the application of an ultrasonic knife, updated endoscopic instruments, and increasingly improved surgical techniques, laparoscopic sigmoid vaginal replacement surgery has gradually replaced the traditional open surgery.

**Fig. 1:**
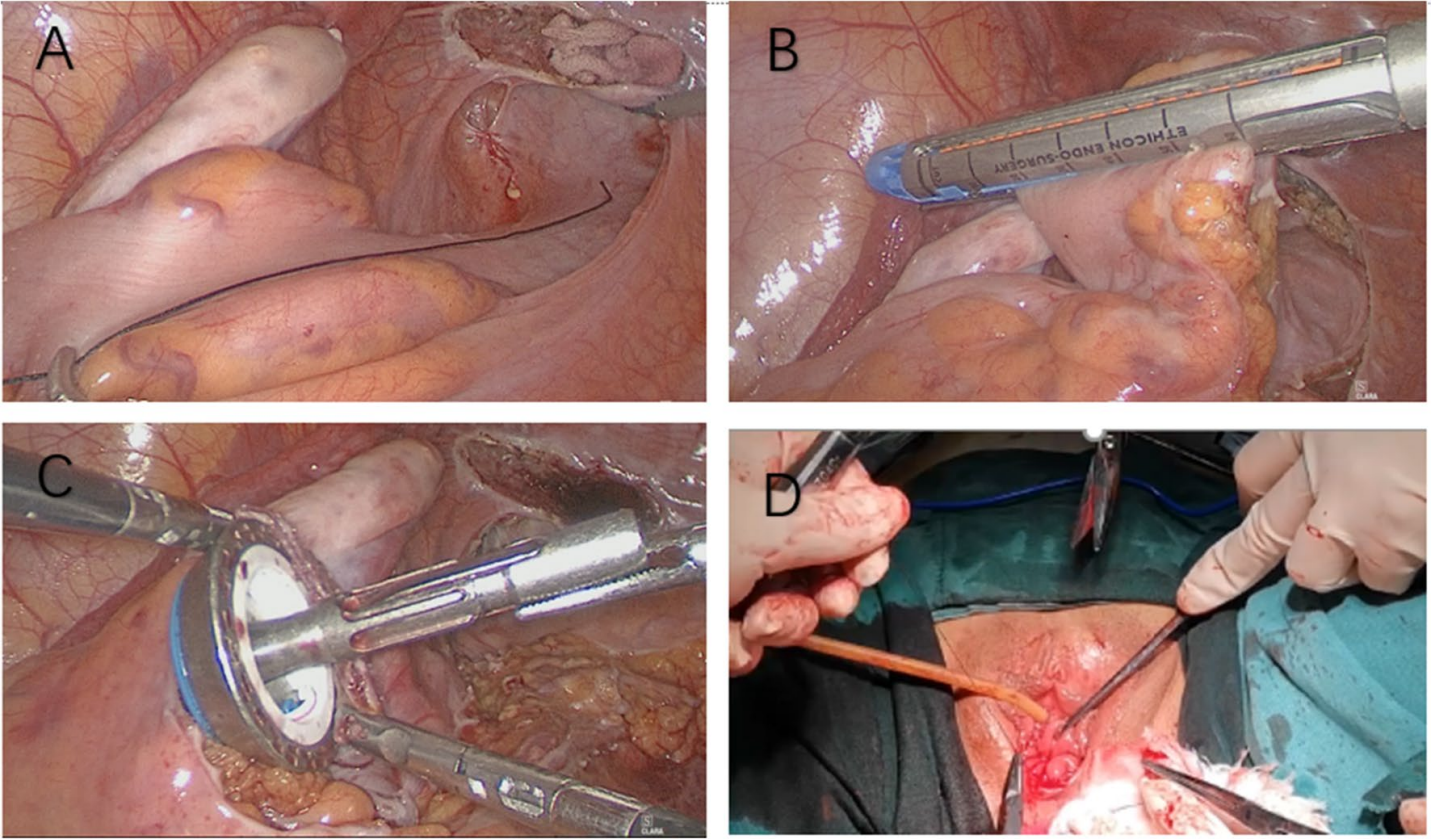
**A–D** Total laparoscopic sigmoid vaginoplasty

At present, the operating technology of sigmoid vaginoplasty has been mature, and the average operation time, blood loss, complications, and post-operative complications reported in the literature are low, so it is a good surgical method [11, 12]. Selecting the grafted intestinal segment with sufficient length and a good blood supply is necessary, and the end-to-end anastomosis of the sigmoid colon under laparoscopy is of great concern. The traditional sigmoid colon anastomosis method is to pull the sigmoid colon out from the abdominal or vaginal cavity. Before that, we need to test the mesenteric vascular tension of the descending colon distal end to pull the descending colon distal end smoothly. We need to fully open the peritoneum of the sigmoid colon as far as possible so as to free the inferior mesenteric vessels and to facilitate pulling the distal end without tension. However, sometimes, even if the submesenteric blood vessels are fully dissociated, it is still difficult to pull the distal end of the descending colon out of the cavitation, so it is necessary to pull it out of the abdominal wall incision to avoid injury. In our hospital, the sigmoid colon was pulled out of the abdominal wall for anastomosis at an early stage. The right lower abdominal incision was required to be extended by 2.5–3 cm. In a recent case of sigmoid transvaginal surgery, we optimized the laparoscopic incision so that the abdominal incision was no longer lengthened. Still, instead, the drill was inserted through the vaginal cavitation. The drill was inserted into the broken end of the intestine under the laparoscope and sutured, all other steps being the same as in traditional surgery, further reducing the risk of traction (Fig. 1). What needs to be improved is the technique of laparoscopy. Nowadays, with the improvement of laparoscopic technology, this does not prolong the operation time or increase the risk of infection, so it is worth popularizing. Some hospitals have started to use the single-hole operation, which requires further equipment, with single-hole instruments, single-hole endoscopic techniques, and surgical techniques, and there is still a risk of poor navel healing [13]. Our optimization of this operation is based on the incision of the traditional laparoscopic surgery, which no longer lengthens the incision, does not need training in the single-hole technique, makes full use of the natural cavity and vaginal cavity to achieve the same surgical effect, and does not increase the operative time with the skillful operation and suturing skills required for laparoscopic surgery.

The sexual life of the neovagina after the operation is a key problem that deserves our attention. Our post-operative follow-up found that married patients or those with sexual partners reported high sexual satisfaction, good lubrication with drainage, and an acceptable odor range. Considering each mean domain score, those women with MRKH syndrome treated by laparoscopic sigmoid vaginoplasty (and completing the FSFI questionnaire) could be considered “normal” in terms of desire, arousal, lubrication, orgasm, and global sexual satisfaction. Other studies have also reported that sigmoid colonic vaginal replacement is associated with better sexual satisfaction [6, 7, 14]. Although overall satisfaction with sexual activity was high, there were limitations because the scores of various indicators for women in sexual activity were not separately listed and compared.

For vaginal replacement surgery, in addition to the sigmoid colon method, peritoneal vaginal replacement is also a common way of operation [2, 14]. Still, it takes a long time to use the vaginal mold after the operation. The width and depth of the artificial vagina are not enough, which may affect sexual satisfaction. There is also a risk of bladder or rectal injury and rectal–vaginal fistula [15]. Davydov’s laparoscopic neo-vaginoplasty is also an alternative way to treat MRKH. It showed a shorter operation time with relatively more post-operative complications (19.0%) [2]. Robotics-assisted surgery is also feasible [16], but it increases the economic burden of patients and it is not conducive to the promotion of its wide use in hospitals. In recent years, vaginoplasty using biological mesh [17–19] has also appeared. However, the high price of biological mesh limits its wide use, and its long-term efficacy and mesh-related complications still need to be further tracked (Table 3).

**Table 3:** Surgical outcome of laparoscopic sigmoid vaginoplasty: comparison with other series

References	Cases	Surgery type	Conversion rate (%)	Operative time (median or mean, range, min)	Blood loss (median or mean, range, ml)	Intra-operative complications (%)	Post-operative complications (%)
Zhang et al. [11]	11	Total laparoscopic sigmoid vaginoplasty	0	187 ± 19	132 ± 24	0	4 (36.4%) (1 vaginal opening stenosis)
Wang et al. [12]	10	Total laparoscopic sigmoid vaginoplasty	0	108.4 ± 52.6 (130–210)	149.2 ± 54.8 (60–170)	0	1 (10.0%) (incision infection and neovaginal stenosis)
Baruch et al. [15]	21	Davydov’s laparoscopic neo-vaginoplasty	0	79 ± 55	–	2 (9.5%)	4 (19.0%)
Zhu et al. [18]	53	Vaginoplasty using tissue-engineered biomaterial mesh	0	26. 9 (20—40)	30–50	0	1 (1.9%)
Qin et al. [2]	620	Laparoscope-assisted peritoneal vaginoplasty techniques	5 (0.8%) needed sigmoid vaginoplasty	Luohu-1 105.0 (83–210); Luohu-2 171 (45–220)	80.50 (20–200); 30.25 (20–100)	16 (2.6%) (16 rectal injury)	9 (1.5%) (9 rectal–vaginal fistula)

In addition to MRKH syndrome, laparoscopic sigmoid vaginoplasty should also be used in international sex-change surgery and has received satisfying results [20]. At present, most literature indicates that patients’ sexual satisfaction and sexual life index scores after sigmoid vaginoplasty are better than those after use of other surgical methods [6, 7, 14]. The sigmoid vaginal replacement method reported by our center had no intra-operative or post-operative complications, and the patients who were followed up showed that the surgical effect was also highly satisfactory. We believe that laparoscopic sigmoid vaginoplasty conducted by skilled surgeons in an endoscopic center is worthy of further promotion.

## Conclusion

Laparoscopic sigmoid vaginoplasty can achieve the goal of making a functional neovagina. The main advantages of this surgical technique are that it is minimally invasive and has fewer complications post-operation. It is a good procedure for patients with MRKH syndrome.

## Data Availability

All data generated or analyzed during this study are included in this article.
